# Modified Essex-Lopresti procedure with percutaneous calcaneoplasty for comminuted intra-articular calcaneal fractures: a retrospective case analysis

**DOI:** 10.1186/s12891-018-1995-9

**Published:** 2018-03-09

**Authors:** Jen-Ta Shih, Chun-Lin Kuo, Tsu-Te Yeh, Hsain-Chung Shen, Ru-Yu Pan, Chia-Chun Wu

**Affiliations:** Department of Orthopaedic Surgery, Tri-Service General Hospital, National Defense Medical Center, No. 325, Sec. 2, Chenggong Rd., Neihu Dist, Taipei City, 11472 Taiwan, Republic of China

**Keywords:** Modified Essex-Lopresti, Minimally invasive, Calcaneoplasty, AOFAS ankle score, Calcaneal fractures

## Abstract

**Background:**

The ideal treatment for comminuted intraarticular calcaneal fractures is still debated. Open reduction and internal fixation (ORIF) is the most popular surgical procedure; however, wound complications, implant choice, and infection remain major concerns. This study aimed to demonstrate the results of an innovative, minimally invasive surgical procedure, namely, a closed reduction technique using large-diameter Steinmann pins and percutaneous calcaneoplasty using injectable calcium sulfate cement (MIIG X3, Wright Medical Technology, Inc., Arlington, TN), in patients with comminuted calcaneal fractures.

**Methods:**

From January 2012 to January 2014, 20 patients (three women, 17 men) with comminuted calcaneus fractures (Sanders classification type III and Essex-Lopresti classification joint-depression type fracture) were included. Plain films and CT scans were obtained preoperatively in all patients. The operation was performed within three days post-injury, and patients were not allowed to bear weight until three months postoperatively. During this period, the patients were educated on how to perform bed exercises for joints above the surgical site, including muscle strengthening and body conditioning. Early active range of motion exercises for the ankle and forefoot began 3 to 6 weeks postoperatively. All patients were followed up regularly. The results were assessed using the American Orthopaedic Foot and Ankle Society (AOFAS) ankle score and Böhler’s angle of the calcaneus.

**Results:**

After an average follow-up of two years, none of the patients required further surgery or experienced soft tissue complications. The clinical results were rated good to excellent on the AOFAS scale in 80% of the cases (16 of 20 patients), and most patients had pain relief and returned to their former daily activities at the same level as before the injury.

**Conclusions:**

A modified Essex-Lopresti procedure with percutaneous calcaneoplasty appears to be a safe and effective procedure to treat comminuted calcaneal fractures with acceptable functional results. Long-term outcomes and additional cases using this technique are required to support our conclusion.

## Background

Calcaneal fractures account for approximately 2% of all fractures, and displaced intra-articular fractures comprise 60% to 75% of these injuries [1]. These fractures increase the calcaneal width, involve the subtalar joint surface, and decrease Böhler’s angle. Controversy still exists regarding their classification, treatment, and postoperative management, as well as the appropriate operative technique for this type of fracture; therefore, calcaneal fractures remain among the most challenging fractures for orthopaedic surgeons to treat. Closed reduction with percutaneous pinning, a minimally invasive technique, is believed to result in fewer soft tissue injuries and fewer wound complications [2]. Indications for this technique include the following: (a) Sanders 2C tongue-type fractures; (b) displaced calcaneal tuberosity fractures; (c) temporary stabilization of fractures with severe soft tissue compromise; and (d) fractures in patients with relative contraindications to open surgery. We have been using modified Essex-Lopresti reduction with percutaneous pinning and percutaneous calcaneoplasty with a minimally invasive injectable graft (MIIG) to augment treatment for comminuted calcaneal fractures since 2012 and herein describe our first 20 consecutive cases with an average follow-up of two years. The aim of the study was to demonstrate the clinical efficacy and safety of a minimally invasive surgical technique for comminuted calcaneal fractures that resolves the complications resulting from traditional ORIF.

## Methods

**Fig. 1:**
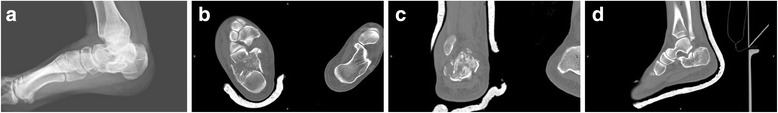
Calcaneal fracture in a 61-year-old man. **a** Preoperative plain film demonstrating a nearly negative Böhler’s angle, joint depression type; **b** CT scan demonstrating a Sanders type III fracture and increased calcaneal width in the axial plane, **c** coronal plane, and **d** sagittal plane

The inclusion criteria were as follows: cases conforming to the diagnostic criteria for unilateral closed intra-articular calcaneal fractures and CT and X-ray examinations confirming a Sanders type III fracture and an Essex-Lopresti joint-depression type fracture (Fig. 1). Paediatric patients, patients with psychiatric disorders or accompanying severe trauma requiring another surgery, and patients with open fractures were excluded from the study. Informed consent was obtained from all eligible patients. Approval was obtained from the Institutional Review Board of Tri-Service General Hospital, National Defense Medical Center (approval number: 1–101–05-008). Chart data were analysed for patient demographics and surgical treatment details from the time of injury to the most recent follow-up. The American Orthopaedic Foot and Ankle Society (AOFAS) ankle/hindfoot score was utilized for clinical evaluation, with results graded as excellent (90), good (80), fair (70), and poor (< 70). Böhler’s angle was calculated to assess bone reduction [3].

### Operative technique

**Fig. 2:**
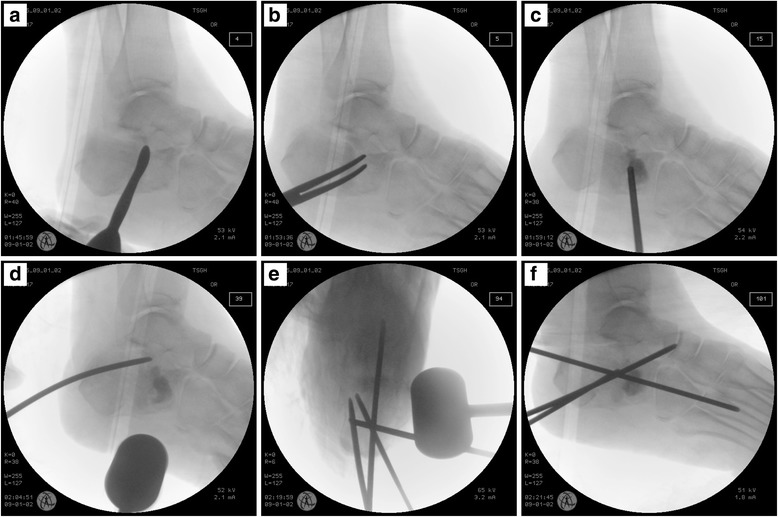
Intraoperative images from an iso-centric image intensifier demonstrating reduction techniques and calcaneoplasty procedures. **a** A dull elevator was inserted through a small incision to elevate the articular surface; **b** a Kelly clamp was used to spread the fracture site; **c** MIIG was directly injected through a trocar to the fracture site; **d** a 3.2-mm Steinmann was applied as a joystick to perform the Essex-Lopresti manoeuvre; **e** Harris axial view demonstrating manipulation with a hammer and correction of the calcaneal axis using the distraction technique with Steinmann pins; and **f** fixation with Steinmann pins across joints to maintain the reduction

Patients were treated with minimally invasive manipulative reduction using large-diameter (3.2–4.0 mm) Steinmann pin fixation. The procedures were performed with patients in the lateral decubitus position under spinal anaesthesia. First, the heel was squeezed to reduce the calcaneal morphology, and the manipulation was performed continually during the operation. Guided by fluoroscopy, the first Steinmann pin was inserted from the superior external side of the calcaneal tuberosity and advanced to the forefoot without crossing the fracture line. With the forefoot plantarflexed, the Steinmann pin was pulled and poked toward the plantar foot, and reduction was performed to allow for calcaneal inversion and eversion as well as flexion and extension of the ankle. We utilized an iso-centric image intensifier intraoperatively to check lateral, Broden’s and Harris axial views of the heel and thus confirmed that Böhler’s angle was restored within the range of 20°–40°. The length and height of the calcaneus were restored as much as possible. The Steinmann pin was inserted into the tarsal bones after restoring the subtalar joint surface. A second Steinmann pin was inserted inferior to the first Steinmann pin and in the direction parallel to the inferior calcaneus for fracture fragment stabilization as well as fixation and support between the tarsal bones and the calcaneus. If necessary, a third Steinmann pin was inserted from the posterior of the calcaneus, traversing the fractured fragments of the subtalar joint and reaching the tarsal bones. We made a minimal incision through the modified sinus tarsi approach to elevate the articular surface, spread the fractured site, and remove blood clots with a suction tip under fluoroscopic guidance before the calcaneoplasty (Fig. 2). The augmentation was completed by the percutaneous injection of 10 ml of calcium sulfate cement (MIIG® X3, Wright Medical Technology, Inc., Arlington, TN) into the fractured site through a trocar. Cast immobilization was applied postoperatively, and protected weight-bearing ambulation was advised within 12 weeks. The pins were draped with antibiotic-coated dressings and were removed after 12 weeks in case of pin tract infection [4].

## Results

**Fig. 3:**
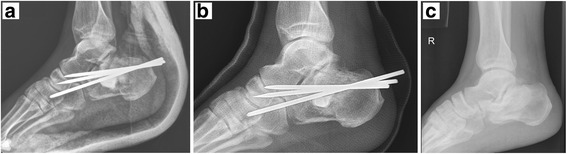
Calcaneal fracture in a 61-year-old man (continued). **a** A postoperative plain film demonstrating restoration of Böhler’s angle. **b** A postoperative plain film two months postoperatively. **c** A reduction of Böhler’s angle was noted 20 months later

We retrospectively reviewed 20 patients (three women and 17 men) with a mean age of 50 years (range, 31 to 66 years) who sustained comminuted intra-articular calcaneal fractures and were treated with closed reduction and percutaneous pinning combined with MIIG augmentation at our institution between January 2012 and January 2014. The injury mechanism was a fall from a height on hard ground in 13 cases and motor vehicle accidents in the remaining cases. Preoperatively, conventional X-ray and CT scan reconstructions were obtained in all cases. Surgery was scheduled within three days because the success of manipulative reduction is correlated with early surgery and minimal soft tissue violation from the surgical technique. All cases progressed to bony union within 3 months. According to the AOFAS score, clinical results were excellent in 6 cases, good in 10 cases, and fair in 4 cases. The mean preoperative Böhler’s angle was − 1.8° (− 16.1°–16.6°), while the mean postoperative value was 25.9° (20.8°–33.3°) (normal Böhler’s angle is between 20° and 40°; Table 1). After an average follow-up of 2 years, the mean Böhler’s angle of the injured calcaneus was 21.7° (17.4°–28.8°) (Fig. 3). Most patients returned to normal activities of daily living after 12 weeks or later. No wound complications or adverse reactions were observed.

**Table 1:** Patient data

Patient	Sex	Preop. BA	Postop. BA	Follow-up BA	Follow-up (months)	AOFAS score	Aetiology
1	M	−16.1	20.8	19.9	31	80	MVA
2	M	−11.8	22.3	18.7	33	81	Tumble
3	M	1.3	33.3	19.0	17	80	Tumble
4	F	−10	21.7	20.4	18	75	MVA
5	M	−2.5	26.6	21.4	31	87	MVA
6	M	0	22.5	21.2	26	90	Tumble
7	M	16.6	24.6	21.5	22	97	Tumble
8	M	−7.2	24.1	19.5	24	82	Tumble
9	M	13.8	25.8	21.9	15	93	Tumble
10	M	10.9	22.8	18.1	28	91	Tumble
11	F	9.0	31.1	24.5	16	78	MVA
12	M	−14.3	21.0	17.4	26	70	MVA
13	M	−15.9	30.1	24.0	22	86	Tumble
14	M	1.2	32.7	28.8	27	88	Tumble
15	M	3.2	28.9	26.4	21	86	Tumble
Patient	Sex	Preop. BA	Postop. BA	Follow-up BA	Follow-up (months)	AOFAS score	
16	M	−7.8	24.8	22.8	30	92	Tumble
17	M	−11.6	23.2	19.6	23	76	Tumble
18	M	−3.9	25.9	22.4	19	84	Tumble
19	F	1.7	24.8	18.2	26	82	MVA
20	M	7.5	30.6	28.5	22	94	MVA
Average		−1.8°	25.9°	21.7°	23.9	84.6	

*BA* Böhler’s angle, *AOFAS* American Orthopaedic Foot and Ankle Society, *MVA* Motor vehicle accident

## Discussion

Most calcaneal fractures are high-energy injuries that are accompanied by various degrees of soft tissue damage. Open reduction using an extensile lateral approach has traditionally been used to treat displaced intra-articular calcaneal fractures, and the surgery is generally delayed until soft tissue swelling has subsided. The major complication associated with open reduction is wound complications, including plate exposure and skin edge necrosis. Despite the presence of advanced surgical techniques and more favourable designs and implant materials, the prevalence of wound complications remains high in open reduction [5]. Essex-Lopresti [6] described a percutaneous reduction manoeuvre several decades ago that was successfully performed by Tornetta [7] in 41 patients with tongue type IIC calcaneal fractures. The purpose of the manoeuvre is to disimpact the fracture by lifting one of the pins and simultaneously plantar flexing the foot. If reduction of the tongue fragment is successful, the second pin is advanced across the anterior process. Definitive fixation is achieved with a large cannulated lag screw that is inserted over the guide wire through small stab incisions placed around the guide pins. Currently, this minimally invasive surgical technique has become widely employed. The indications were limited to Sanders 2C tongue-type fractures, displaced calcaneal tuberosity fractures, and fractures in patients who are not amenable to open surgery. The minimally invasive reduction and fixation technique in the present study focused on treating complicated calcaneal fractures that are classified as Sanders type III joint depression fractures, which are considered to be one of the most challenging types of calcaneus fractures.

Calcaneal morphology is essential for hindfoot and ankle function, and fracture reduction helps to restore a congruent subtalar joint. Agren et al. [8] conducted a prospective, randomized, controlled multicentre trial to compare operative with nonoperative treatments of displaced intra-articular calcaneal fractures. Eighty-two patients were included. Operative treatment, despite increasing the risk of complications, appeared to have some benefits at eight to twelve years, including a trend towards increased patient-reported primary visual analogue scale (VAS) scores for pain and function and a reduced prevalence of posttraumatic arthritis evident on follow-up radiographs. Once poor calcaneal morphology and late post-traumatic arthritis develop, they may lead to persistent functional impairment requiring further treatment, such as subtalar arthrodesis [9]. The correlation between Böhler’s angle and clinical outcomes in displaced intra-articular calcaneal fractures has been demonstrated [10]. In other words, restoration of hindfoot morphology may be more important than the anatomical reduction of the posterior facet to achieve good clinical results in comminuted calcaneal fractures [11]. The surgical outcomes in our study demonstrated that minimally invasive manipulative reduction and percutaneous pinning can restore hindfoot morphology, with a mean increase in Böhler’s angle of 27.7°. A mean loss of 4.2° at 2 years after the trauma was observed; however, the functional results remained satisfactory.

Traditionally, operative treatment for fractures should be performed within three weeks after the injury prior to early consolidation of the fracture; nevertheless, delaying definitive fixation of closed, intra-articular calcaneus fractures did not decrease wound complication rates when using the extensile lateral approach [12]. The time from injury to surgery in our study was three days or less. The benefits of our minimally invasive technique include the accessibility of manipulative reduction with early surgery and a low incidence of wound complications.

Stulik et al. [13] performed surgery within 48 h in 89% of the patients in their series and stated that earlier surgery facilitated reduction. However, the risk of deep infection and potential limb-threatening complications following open reduction and internal fixation of calcaneal fractures was significant [5]. deWall et al. [14] described a deep infection rate of 14.3% and a minor wound complication rate of 21.4% in 41 patients treated with open reduction via an extensile lateral approach, with no deep infections and a 6% minor wound complication rate in 79 patients who underwent percutaneous treatment. A relatively short operative time and less wound surface exposure may have contributed to these results.

Most injectable bone substitutes are calcium phosphate compounds, calcium sulfate compounds, or both. Following mixing, bone substitute pastes can be injected into a fracture space for augmentation as an alternative to a bone graft, and the mechanical strength of these pastes when compressed resembles that of cancellous bone [15]. Early clinical results have shown a reduced time to full weight-bearing when bone substitutes have been used for augmentation of calcaneal fractures [16]. Injectable calcium sulfate bone substitutes (MIIG® X3, Wright Medical Technology, Inc., Arlington, TN) were used for all patients in the present study. There was no increased risk of infection, and these substitutes provided more convenience during the operation. In fact, the use of autologous bone grafts has not been shown to have radiological or functional benefit [17]. Biggi et al. [18] described a percutaneous calcaneoplasty technique using balloon reduction and augmentation with injectable bone substitutes of calcium phosphate or cement, and this technique demonstrated good clinical results for treating calcaneal fractures without internal fixation.

Traditional extensile open surgery is technically demanding, and there is an extensive learning curve for surgeons who intend to achieve anatomic reduction and provide absolute stability for comminuted calcaneal fractures, as well as a prolonged operative time and extensive wound exposure. A minimally invasive technique achieves functional reduction, provides relative stability, minimizes soft tissue dissection, preserves the blood supply and, thus, may be more suitable given the characteristics of comminuted calcaneal fractures. It also offers an alternative approach in patients who have significant medical co-morbidities (smokers, diabetics, and patients with peripheral vascular disease or intolerance to prolonged surgery), in whom open reduction would be hazardous and non-operative treatment would be inadequate [19]. In fact, most surgeons learn the essentials of minimally invasive techniques earlier than previously thought [20]. Although this is a retrospective study with a limited number of cases and a two-year follow-up, the results are encouraging, suggesting that true minimally invasive percutaneous stabilization is an alternative for the surgical treatment of such challenging fractures.

## Conclusion

To our knowledge, a minimally invasive technique combining a modified Essex-Lopresti manoeuvre and percutaneous calcaneoplasty with injectable bone substitutes of calcium sulfate has not been previously described in the literature. This technique can be performed in the presence of soft tissue swelling immediately following trauma, with a low incidence of wound infection/dehiscence. Although anatomic reduction of the articular surface cannot be achieved, this technique restores the calcaneal morphology by improving Böhler’s angle and results in acceptable functional outcomes without major complications in patients with Sanders type III joint depression type calcaneal fractures. Further study of long-term outcomes and additional cases using this technique is required to support our conclusion.

## Abbreviations

AOFAS: American Orthopaedic Foot and Ankle Society
MIIG: Minimally invasive injectable graft
ORIF: Open reduction and internal fixation
